# Analysis of *Salmonella enterica* Serotype Paratyphi A Gene Expression in the Blood of Bacteremic Patients in Bangladesh

**DOI:** 10.1371/journal.pntd.0000908

**Published:** 2010-12-07

**Authors:** Alaullah Sheikh, Richelle C. Charles, Sean M. Rollins, Jason B. Harris, Md. Saruar Bhuiyan, Farhana Khanam, Archana Bukka, Anuj Kalsy, Steffen Porwollik, W. Abdullah Brooks, Regina C. LaRocque, Elizabeth L. Hohmann, Alejandro Cravioto, Tanya Logvinenko, Stephen B. Calderwood, Michael McClelland, James E. Graham, Firdausi Qadri, Edward T. Ryan

**Affiliations:** 1 International Centre for Diarrhoeal Disease Research, Bangladesh (ICDDR,B), Dhaka, Bangladesh; 2 Division of Infectious Diseases, Massachusetts General Hospital, Boston, Massachusetts, United States of America; 3 Department of Medicine, Harvard Medical School, Boston, Massachusetts, United States of America; 4 Department of Microbiology and Immunology, University of Louisville School of Medicine, Louisville, Kentucky, United States of America; 5 Vaccine Research Institute of San Diego, San Diego, California, United States of America; 6 Division of Biostatistics, Institute for Clinical Research and Health Policy Studies (ICRHPS), Tufts Medical Center, Boston, Massachusetts, United States of America; 7 Department of Microbiology and Molecular Genetics, Harvard Medical School, Boston, Massachusetts, United States of America; 8 Department of Immunology and Infectious Diseases, Harvard School of Public Health, Boston, Massachusetts, United States of America; Instituto Butantan, Brazil

## Abstract

**Background:**

*Salmonella enterica* serotype Paratyphi A is a human-restricted cause of paratyphoid fever, accounting for up to a fifth of all cases of enteric fever in Asia.

**Methodology/Principal Findings:**

In this work, we applied an RNA analysis method, Selective Capture of Transcribed Sequences (SCOTS), and cDNA hybridization-microarray technology to identify *S*. Paratyphi A transcripts expressed by bacteria in the blood of three patients in Bangladesh. In total, we detected 1,798 *S*. Paratyphi A mRNAs expressed in the blood of infected humans (43.9% of the ORFeome). Of these, we identified 868 in at least two patients, and 315 in all three patients. *S*. Paratyphi A transcripts identified in at least two patients encode proteins involved in energy metabolism, nutrient and iron acquisition, vitamin biosynthesis, stress responses, oxidative stress resistance, and pathogenesis. A number of detected transcripts are expressed from PhoP and SlyA-regulated genes associated with intra-macrophage survival, genes contained within *Salmonella* Pathogenicity Islands (SPIs) 1–4, 6, 10, 13, and 16, as well as RpoS-regulated genes. The largest category of identified transcripts is that of encoding proteins with unknown function. When comparing levels of bacterial mRNA using *in vivo* samples collected from infected patients to samples from *in vitro* grown organisms, we found significant differences for 347, 391, and 456 *S*. Paratyphi A transcripts in each of three individual patients (approximately 9.7% of the ORFeome). Of these, expression of 194 transcripts (4.7% of ORFs) was concordant in two or more patients, and 41 in all patients. Genes encoding these transcripts are contained within SPI-1, 3, 6 and 10, PhoP-regulated genes, involved in energy metabolism, nutrient acquisition, drug resistance, or uncharacterized genes. Using quantitative RT-PCR, we confirmed increased gene expression *in vivo* for a subset of these genes.

**Conclusion/Significance:**

To our knowledge, we describe the first microarray-based transcriptional analysis of a pathogen in the blood of naturally infected humans.

## Author Summary

*Salmonella enterica* serotype Paratyphi A is a significant and emerging global public health problem and accounts for one fifth of all cases of enteric fever in many areas of Asia. *S*. Paratyphi A only infects humans, and the lack of an appropriate animal model has limited the study of *S.* Paratyphi A infection. In this study, we report the application of an RNA analysis method, Selective Capture of Transcribed Sequences (SCOTS), to evaluate which *S*. Paratyphi A genes are expressed directly in the blood of infected humans. Our results provide insight into the bacterial adaptations and modifications that *S.* Paratyphi A may need to survive within infected humans and suggest that similar approaches may be applied to other pathogens in infected humans and animals.

## Introduction

*Salmonella enterica* serotype Paratyphi A (*S*. Paratyphi A) is an emerging food and water-borne pathogen that currently accounts for approximately 1 in 5 cases of enteric fever in South Asia [1], [2]. Although previously thought to cause a milder illness than *Salmonella enterica* serotype Typhi (*S.* Typhi), recent studies suggest that *S.* Paratyphi A causes a clinical syndrome quite similar to that caused by *S.* Typhi [1], [3]. Over the past decade, *S.* Paratyphi A isolation rates have increased throughout South Asia, along with antimicrobial resistance [1], [3]–[6]. The reasons for this emergence of *S.* Paratyphi A are unclear, and may relate in part to secondary effects of vaccine programs targeting *S.* Typhi [6]. No commercially available vaccine protects against *S.* Paratyphi A infection, although some protection against *S*. Paratyphi B is provided by the oral live attenuated typhoid vaccine strain Ty21a (a derivative of *S*. Typhi wild type strain Ty2) [1], [7], [8]. Increasing rates of infection, the lack of a commercially available vaccine effective against *S*. Paratyphi A infection, and steadily increasing resistance of *S*. Paratyphi A to antimicrobial agents make *S*. Paratyphi A infection a growing public health concern.

Following oral ingestion, *S*. Paratyphi A organisms invade intestinal epithelial cells, are taken up by gut-associated lympho-reticular tissues, and enter the systemic circulation. In the bloodstream, a majority of organisms reside within professional phagocytic cells, while the remainder are extracellular [9]. *S*. Paratyphi A studies have been limited by both a lack of an adequate animal model, and the low number of microorganisms present in the blood of infected individuals (estimated at 0.1–10,000 *Salmonella* organisms per ml of blood) [9]–[11]. Most of what is known about the pathogenesis of *S*. Paratyphi A has, therefore, been extrapolated from studies with *S.* Typhi and *S.* Typhimurium, and comparative genomic analyses [12]–[21].

To begin to address pathogen-host interactions during this unique human-restricted infection, we applied a transcript capture and amplification technique, Selective Capture of Transcribed Sequences (SCOTS) [22], along with microarray technology, to assess whether we could detect *S*. Paratyphi A mRNA directly in the blood of bacteremic patients. To identify genes whose expression might be potentially regulated *in vivo*, we also compared the relative level of bacterial genes detected *in vivo* to those detected using the same technique on *in vitro* grown organisms. We then used RT-qPCR to validate expression of genes detected in our screen.

SCOTS has previously been used in gene expression studies of a number of organisms, including analyses of *S*. Typhi, *S*. Typhimurium, and *Mycobacterium tuberculosis*, using *ex vivo* human macrophage models [13], [14], [16], [23]. SCOTS has also been applied to evaluate bacterial gene expression in *Helicobacter pylori* in gastric mucosa biopsies of infected humans, and *Hemophilus ducreyi* in pustules of infected humans [24], [25]. To our knowledge, however, no previous study has used this approach to assess bacterial gene expression directly in the blood of infected humans.

## Methods

### Ethics statement

This study was conducted according to the principles expressed in the Declaration of Helsinki. We obtained written consent from each patient prior to participation. Written informed consent was obtained from parents authorizing the participation of their children in the study and assent was obtained from children greater than 5 years old. This study was approved by the ethical and research review committees of the ICDDR,B and the Human Research Committee of Massachusetts General Hospital.

### Study subject selection, sample collection, and recovery of organisms

Individuals (3–59 years of age) presenting to the International Centre for Diarrhoeal Disease Research, Bangladesh (ICDDR,B) Hospital or Dhaka Medical College Hospital with fever of 3–7 days duration (≥39°C), without an obvious focus of infection, and lacking an alternate diagnosis were eligible for enrollment. After obtaining written consent, we collected venous blood (5 ml from children <5 years, and 10 ml from all others), and immediately placed 2 ml of blood into TRIzol (Invitrogen Life Technologies, Carlsbad, CA) at a 1 (blood):2 (TRIzol) volume ratio. We mixed these samples and stored them at −70°C for later analysis. We next cultured 3–5 ml of day 0 blood using a BacT/Alert automated system, sub-culturing positive bottles on MacConkey agar, and identifying isolates using standard biochemical tests and reaction with *Salmonella* specific antisera [26]. Following collection of blood, patients were initially treated with oral ciprofloxacin or cefixime, or injectable ceftriaxone, and antibiotics were continued for up to 14 days at the discretion of the attending physician.

### cDNA synthesis and amplification

To create cDNA of organisms in the blood of bacteremic patients (*in vivo* sample), we used TRIzol preserved blood samples of patients whose day 0 culture subsequently grew *S*. Paratyphi A. To generate corresponding *in vitro* cDNA samples, we grew each patient's bacterial isolate in Luria Bertani (LB) broth until mid-log growth phase (OD_600_ 0.45–0.6), and then immediately placed these samples into TRIzol at a 1 (mid-log culture):2 (TRIzol) volume ratio. We recovered total RNA from TRIzol preserved *in vivo* and *in vitro* samples per manufacturer's instructions (Invitrogen), and treated with DNase I on RNeasy columns (Qiagen). We converted 5 µg of total extracted RNA into cDNA using random priming (T-PCR) to obtain a representative amplifiable double-stranded cDNA population as described by Froussard *et al.* [27], with modifications as previously described by Graham *et al.* [23]. Briefly, we used Superscript III (Invitrogen) to synthesize first strand cDNA with K9RNA (for *in vivo* sample) or F9RNA (for *in vitro* sample) primers with a defined 5′ end terminal sequence and a random nonamer at the 3′ end (Supplementary Table S1). We then synthesized second strands using the same primers and Klenow fragment (Invitrogen) according to manufacturer's instruction. We then PCR-amplified double stranded cDNA with K9 primer lacking random terminal residues for *in vivo* samples, and F9 primer for *in vitro* samples for a total of 30 cycles (94°C for 1 minute, 55°C for 1 minute, 72°C for 45 second cycle, with an initial denaturation at 94°C for 2 minutes).

### Genomic DNA biotinylation and rRNA blocking-plasmid construction

SCOTS requires capturing bacterial cDNA from a mixture of host and microbial nucleic acid by solution-phase hybridization to biotinylated bacterial genomic DNA (gDNA) [23]. To generate gDNA, we grew one of the *S*. Paratyphi A clinical isolates to mid-logarithmic growth phase in LB at 37°C, and purified gDNA using Easy DNA (Invitrogen). We then biotinylated extracted gDNA with photobiotin acetate (Sigma), and sonicated as previously described [22]. To minimize capture of cDNA of ribosomal RNA by biotinylated gDNA, we blocked ribosomal RNA encoding sequences in the gDNA by pre-hybridizing with sonicated DNA fragments from plasmids encoding 16S and 23S cognate rRNA. To generate these plasmids, we PCR-amplified *S*. Paratyphi A 16S and 23S rRNA genes, and cloned these products into pKK223-3 to generate pRibDNA_PTA.

### SCOTS

We performed three rounds of SCOTS on *in vivo* and *in vitro* cDNA samples, separately, as previously described [23]. Briefly, we mixed denatured biotinylated *S*. Paratyphi A gDNA with blocking pRibDNA_PTA, and added this denatured mixture to cDNA samples, hybridizing samples overnight at 67°C. We captured biotinylated gDNA-cDNA hybrids using streptavidin-coated magnetic beads (Dynal M-280). After washing samples, we eluted captured cDNA with NaOH, PCR-amplified *in vivo* and *in vitro* cDNA samples with K9 or F9 primers, respectively, and purified products using Qiagen PCR column purification kits. We performed three hybridization and amplification cycles to obtain bacterial cDNAs for microarray hybridization.

### cDNA hybridization-microarray analysis

We differentially labelled *in vivo* and *in vitro* SCOTS-cDNAs for each of the three patients with *S*. Paratyphi A bacteremia [28], and added these products to activated Salmonella ORF microarray glass slides (version STv7S; McClelland Laboratory, Vaccine Research Institute of San Diego, CA, http://www.sdibr.org/Faculty/mcclelland/mcclelland-lab), as previously described [13], [29]. Microarrays contained gene-specific PCR-products of 4,271 ORFs from *Salmonella enterica* strains including ≥97% identical orthologues of more than 95% of annotated *S*. Paratyphi A SARB42 genes. We performed labeling and hybridization in duplicate, with dye reversals. We quantified signal intensities using ScanArray software (ScanArray express, version 3.0.1). To assess potential biases in identifying potentially differentially *in vivo*-expressed genes, we also probed slides comparing SCOTS-product generated from *in vivo* samples to labeled cDNA from *in vitro* samples to which SCOTS was not applied. Additionally, we directly compared *in vitro* cDNAs slides to which SCOTS was and was not applied using analyses described below and similar to Faucher *et al.* [13], which confirmed that no significant biases were introduced using this approach.

For analyses, we subtracted local background from spot signal intensities, and considered a cDNA for an ORF detected in a particular sample if it met the following criteria: 1) the median signal intensity of at least 2 of its 3 replicate spots on the array was at least ten median absolute deviations greater than the median of spots on the microarray corresponding to genes absent from the *S.* Paratyphi A genome, and 2), this criterion was met on greater than 75% of slides for that subject. We then evaluated differences in expression in *in vitro* versus *in vivo* grown organisms for all genes detected in *in vivo* samples. Using LOESS-normalized, log-transformed data, we employed repeated measures ANOVA (to within slide replicate spots) with fixed type (*in vivo* versus *in vitro*) and dye effects with Benjamini-Hochberg correction. We only considered array features with a coefficient of variation in signal intensity of less than 50% within an array. We considered significant variations in signal intensity as determined by ANOVA indication of genes potentially differentially expressed *in vivo* versus *in vitro*. We deposited data in the NCBI Gene Expression Omnibus (GEO, www.ncbi.nlm.nih.gov/geo), accessible through GEO accession number GSE22958. Functional classification of genes was based on J. Craig Venter Institute annotations (http://cmr.jcvi.org/tigr-scripts/CMR/CmrHomePage.cgi).

### Quantitative PCR analysis

We selected a subset of genes identified in our microarray analyses for confirmation by quantitative real time PCR (RT-qPCR). We compared steady state mRNA levels in specimens from two of the patients analyzed by SCOTS-array hybridization, and two additional patients bacteremic with *S*. Paratyphi A that were not included in the SCOTS analyses. We compared *in vivo* expression levels to those present in *in vitro* samples using three bench-top culture replicates of the *S*. Paratyphi A isolate from Patient 3 grown to mid-logarithmic phase in LB, as described above. We included in our selection criteria several genes of interest with high baseline signals and fold-changes by SCOTS-array analysis. For comparison, we used SPA3294 (encoding 50S ribosomal protein L5) as a housekeeping gene with no differential detection by SCOTS-array analysis. We had insufficient sample from patient #1 to perform RT-qPCR for all selected genes, and for this individual, we performed RT-qPCR for only three of the six selected genes. We used SuperScript II (Invitrogen) with random hexamers (Sigma), according to manufacturer's instructions to generate cDNA, and performed RT-qPCR analysis using iQ SYBR Green Supermix reagent (Bio-Rad; Hercules, CA) and a CFX96 Real-time PCR detection system (Bio-Rad; Hercules, CA), as previously described [30]. Primers are listed in Table S1. For each sample, we used no-template controls and samples lacking reverse transcriptase as baseline reactions. After initial denaturation at 95°C for 3 min, the RT-PCR cycle was as follows: denaturation at 94°C for 30 seconds (s), extension at 58°C for 30 s, extension at 72°C for 1 minute, followed by a plate read. We repeated the cycle 40 times, set the calculated threshold cycle (C_T_) in the low/linear portion of product curves, and quantified gene copy numbers using pGEM-T Easy-based plasmids (Promega) containing the gene of interest [30]. We calculated control gene copy numbers using plasmid size and A260 readings, and normalized gene copy numbers against cDNA copies of 16S rRNA. We assessed singularity of product species and size by melting curve analysis.

## Results

### Detection of *S*. Paratyphi A mRNA in the blood of infected humans

**Figure 1:**
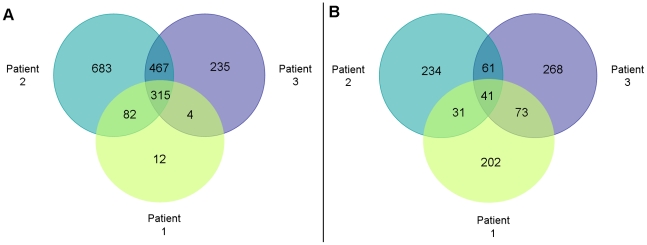
*Salmonella* Paratyphi A genes whose mRNA/cDNA was detected in the blood of infected humans. Venn diagram of (A) number of transcripts of *S*. Paratyphi A genes detected in each patient, and (B) subset with significantly different levels of detection by SCOTS-array comparing *in vivo* to *in vitro* samples.

**Figure 2:**
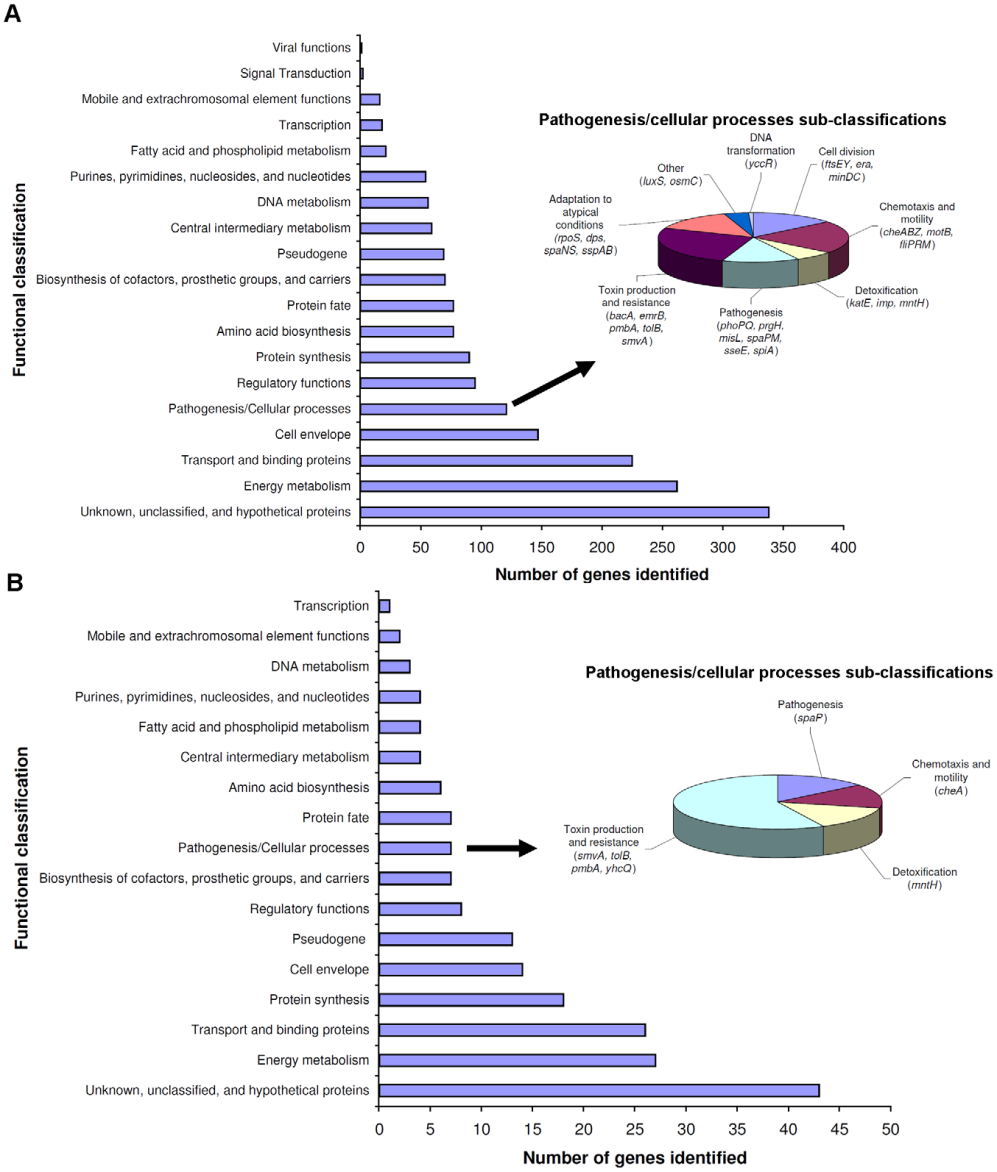
Functional classifications of products of *S*. Paratyphi A genes whose mRNA/cDNA was detected in this study. Functional classification of products of transcripts (A) detected in *in vivo* samples in two or more patients, and (B) associated with significantly different levels of detection in *in vivo* versus *in vitro* samples in two or more patients using SCOTS-array.

We isolated serotype Paratyphi A in the blood of 5 of 89 individuals who met study criteria. In total, we detected transcripts of 1798 *S*. Paratyphi A genes in the blood of infected humans, or 43.9% of ORFs in the *S.* Paratyphi ATCC9150 genome. Of these, we detected 868 transcripts in at least two patients, and 315 in all three patients (Figure 1A and Table S2). Detected transcripts are predicted to encode products that could be categorized into a number of functional groups (Figure 2A). The largest grouping was “unknown/unclassified” (338 transcripts; 18.8% of detected mRNA/cDNAs; 8.3% of *S*. Paratyphi A ORFs). Other large groupings included transcripts of genes associated with pathogenesis (*phoQ*, *rpoS*), nutrient acquisition, energy metabolism, biosynthesis of the essential vitamins biotin (*bioABF*, *thiJ*) and thiamine (*thiC*), iron acquisition (genes in the i*roA* cluster, *fepBDE*, *ybdA*) and stress responses (*htrA*, *groEL*, *groES*, *dnaK*) including oxidative stress resistance (*katE*, *umuCD*). Forty-three of the transcripts corresponded to genes contained within *Salmonella* Pathogenicity Islands (SPIs) 1–4, 6, 10, 13, and 16 (annotated in column E of Table S2).

### Comparison of levels of *S*. Paratyphi A transcripts detected using *in vivo* versus *in vitro* samples

mRNA/cDNA of 11 genes were detected only in *in vivo* samples of at least 2 patients, and not detected in any *in vitro* sample (Table 1). These included transcripts of genes involved in biosynthesis of essential vitamins such as biotin (*bioF*), utilization of alternative carbon sources including ethanolamine (*eut* operon) and propanediol (*pdu* operon), iron acquisition (*fepD*), and resistance to antimicrobial agents (*yegO*, also known as *mdtC*). When considering genes whose expression was detected by SCOTS array in both *in vivo* and *in vitro* samples, we identified transcripts of 910 genes (22.3% of ORFs) with significant and at least 2-fold difference in signal detection between *in vivo* versus *in vitro* samples: 347, 367, and 443 in patients 1, 2, and 3, respectively (Figure 1B and annotated in column Y, AS, and AE of Table S2). We detected transcripts of 206 genes in at least two patients, expression of 194 of which were concordant (4.7% ORFs), and 41 in all three patients (Table 2). We categorized the 194 concordant gene products detected in at least two patients into a number of functional groups (Figure 2B). The largest grouping was “unknown/unclassified” (44 genes). We identified transcripts of 12 genes were located within SPIs (SPI-1, 3, 6, 10, 16) [12], [31]–[34], a number of transcripts corresponded to genes within the PhoP/Q regulatory system involved in intra-macrophage survival [20], [35]–[37], and five genes were in phages unique to *S*. Paratyphi A, including SPA-2 and SPA-3-P2 [12].

**Table 1:** *S.* Paratyphi A (SPA) genes whose transcripts were detected only in *in vivo* samples of at least two patients and not in any *in vitro* sample.

SPA locus	SPA gene	SPA description
SPA0405	eutE	Putative aldehyde dehydrogenase
SPA0612	ccmC	Heme exporter protein C1
SPA0737	yegO	Putative RND-family transporter protein
SPA0826	pduJ	Putative propanediol utilization protein PduJ
SPA1856	cspD	Cold shock-like protein CspD
SPA1957	bioF	8-amino-7-oxononanoate synthase
SPA2142	fepD	Ferric enterobactin transport protein
SPA2483	-	Conserved hypothetical protein
SPA2570	-	Putative phage baseplate assembly protein
SPA2574	-	Putative membrane protein
SPA3456	-	Putative uncharacterized protein

**Table 2:** *S.* Paratyphi A genes whose transcripts had significantly different levels of detection in all three patients comparing *in vivo* to *in vitro* samples using SCOTS/microarray.

Main Role	SPA locus	SPA gene	SPA description	Pt1 FC	Pt 1 pv	Pt2 FC	Pt 2 pv	Pt3 FC	Pt 3 pv
**Pathogenesis/Cellular processes**	SPA0452	*mntH*	Manganese transport protein MntH	3.11	0.0459	2.06	0.0265	2.75	0.0196
	SPA2748	*spaP*	Secretory protein (associated with virulence)	10.93	0.0027	1.36	0.0400	2.03	0.0379
**Amino acid biosynthesis**	SPA0426	*cysM*	Cysteine synthase B	5.39	0.0340	3.66	0.0468	2.24	0.0323
**Biosynthesis of cofactors, prosthetic groups, and carriers**	SPA2972	*yggH*	Conserved hypothetical protein	7.43	0.0027	1.47	0.0445	1.51	0.0335
	SPA4001	*thiC*	Thiamine biosynthesis protein	3.48	0.0099	2.67	0.0253	2.78	0.0496
**Cell envelope**	SPA3474	*yhjN*	Putative polysaccharide biosynthesis protein subunit B	4.46	0.0034	3.99	0.0386	4.25	0.0181
**Central intermediary metabolism**	SPA0545	*nuoK*	NADH dehydrogenase I chain k	9.54	0.0047	5.70	0.0295	3.97	0.0193
**Energy metabolism**	SPA0406	*eutJ*	Putative ethanolamine utilization protein EutJ	7.73	0.0029	7.96	0.0391	3.04	0.0250
	SPA0410	*eutB*	Ethanolamine ammonia-lyase heavy chain	5.07	0.0031	3.05	0.0322	2.40	0.0228
	SPA0828	*pduG*	PduG protein	10.54	0.0030	2.91	0.0244	3.86	0.0220
	SPA3394	*gntR*	Gluconate utilization operon repressor	9.75	0.0027	1.16	0.0403	2.09	0.0206
	SPA3527	*yiaR*	Putative sugar-phosphate isomerase	3.62	0.0446	7.61	0.0363	6.29	0.0497
	SPA3706	*atpA*	ATP synthase alpha subunit	3.76	0.0470	2.90	0.0487	1.03	0.0335
	SPA4097	*nrfD*	Cytochrome c-type biogenesis protein	11.04	0.0034	2.92	0.0256	3.84	0.0284
	SPA4125	*dmsC*	Putative dimethyl sulfoxide reductase subunit C	9.98	0.0026	1.60	0.0495	1.45	0.0317
**Fatty acid and phospholipid metabolism**	SPA1655	*acpP*	Acyl carrier protein	9.57	0.0068	3.04	0.0237	3.05	0.0392
**Hypothetical proteins**	SPA1739	*yccD*	Conserved hypothetical protein	2.87	0.0041	2.45	0.0278	3.82	0.0461
**Mobile and extrachromosomal element functions**	SPA3373	*-*	Conserved hypothetical protein	13.19	0.0037	1.95	0.0248	3.66	0.0326
**Protein fate**	SPA2315	*secF*	Protein-export membrane protein SecF	5.30	0.0030	1.49	0.0292	1.01	0.0348
	SPA2496	*-*	ClpB-like protein	6.25	0.0125	2.27	0.0328	3.09	0.0203
	SPA3012	*hybF*	Hydrogenase-2 component protein	5.51	0.0028	6.58	0.0281	5.56	0.0212
**Protein synthesis**	SPA3288	*rpmD*	50S ribosomal subunit protein L30	7.81	0.0029	4.42	0.0275	3.47	0.0217
	SPA3289	*rpsE*	30S ribosomal subunit protein S5	7.16	0.0042	4.19	0.0276	4.03	0.0196
	SPA3290	*rplR*	50S ribosomal subunit protein L18	5.42	0.0028	4.94	0.0272	4.03	0.0213
	SPA3291	*rplF*	50S ribosomal subunit protein L6	3.70	0.0463	1.98	0.0332	4.53	0.0225
	SPA3304	*rplW*	50S ribosomal subunit protein L23	11.35	0.0036	5.71	0.0429	5.62	0.0273
	SPA3305	*rplD*	50S ribosomal subunit protein L4	13.12	0.0094	3.77	0.0245	2.88	0.0185
	SPA4208	*rpsF*	30s ribosomal protein S6	9.06	0.0025	5.87	0.0234	4.46	0.0205
**Regulatory functions**	SPA0293	*yfhH*	Putative transcriptional regulator	4.78	0.0367	2.40	0.0259	2.81	0.0338
	SPA0905	*yedE*	Putative membrane protein	3.68	0.0056	5.55	0.0294	3.51	0.0261
**Transport and binding**	SPA0056	*oadA*	Oxaloacetate decarboxylase alpha chain	7.21	0.0037	2.45	0.0259	2.38	0.0201
	SPA0918	*yecC*	Putative ABC transport ATP-binding protein	2.43	0.0035	4.01	0.0256	2.64	0.0262
	SPA1531	*celB*	PTS system, cellobiose-specific IIC component	9.19	0.0028	2.88	0.0274	2.39	0.0294
	SPA2035	*kdpA*	Potassium-transporting ATPase A chain	6.80	0.0032	5.58	0.0268	2.95	0.0499
	SPA3536	*mtlA*	Mannitol-specific enzyme II of phosphotransferase system	6.24	0.0035	1.11	0.0285	4.39	0.0272
**Unclassified**	SPA0411	*eutC*	Ethanolamine ammonia-lyase light chain	8.68	0.0217	3.42	0.0295	1.50	0.0450
**Unknown function**	SPA0409	*eutA*	Putative ethanolamine utilization protein EutA	8.48	0.0028	9.61	0.0286	4.76	0.0265
**Pseudogene**	SPA2109	*dpiB*	Sensor kinase DpiB pseudogene	4.64	0.0333	1.85	0.0333	1.75	0.0252
	SPA3223	*-*	Possible membrane transport protein pseudogene	11.35	0.0044	2.08	0.0262	2.80	0.0249
	SPA3638	*uhpC*	Regulatory protein pseudogene	7.43	0.0029	3.61	0.0246	2.09	0.0335
	SPA3755	*rhlB*	Putative ATP-dependent RNA helicase pseudogene	5.03	0.0341	1.43	0.0315	2.18	0.0260

**SPA:** ***S.*** **Paratyphi A.**

**FC: Fold change - ratio of signal intensities (** ***in vivo/in vitro*** **).**

**PV: P value.**

### Quantitative Real Time-PCR analysis

**Figure 3:**
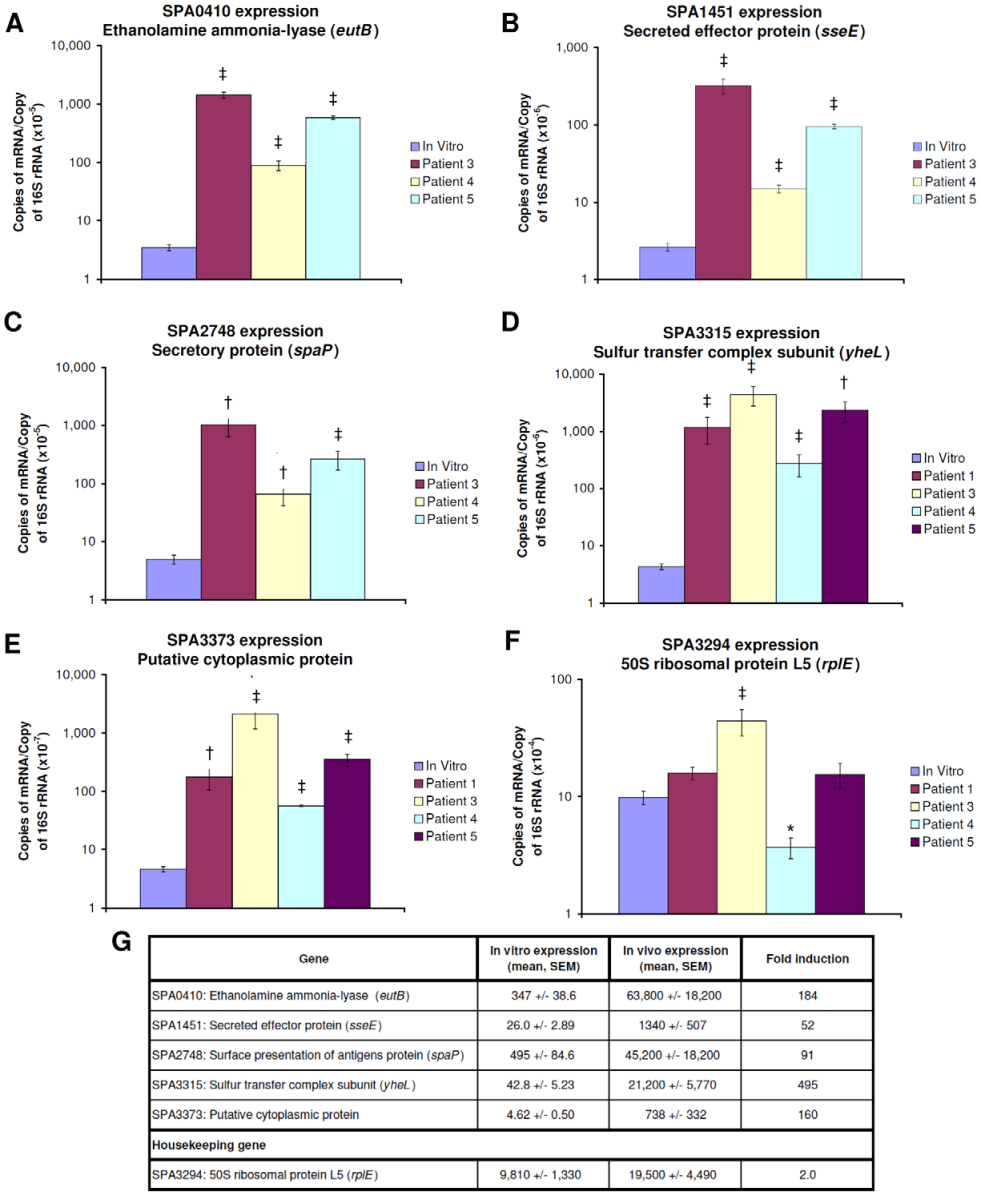
Quantitative-PCR mRNA expression profiles. Quantitative-PCR mRNA expression profiles during *in vitro* and *in vivo* growth of *S*. Paratyphi A of genes associated with significantly different levels of detection in *in vivo* versus *in vitro* samples using SCOTS-array analysis. Quantitative RT-PCR gene profiles of *S.* Paratyphi A genes (A–E) comparing RNA recovered from blood of bacteremic patients to *in vitro* cultures, and a house-keeping gene with no differential expression by SCOTS-microarray analysis (F). Mean copies of mRNA per copy of 16S rRNA, standard error of the mean (SEM), and fold-induction (G) are presented. _*_ p<0.05; † p<0.01; ‡ p<0.001.

To further analyze expression levels of genes identified in our screen, we used quantitative RT-PCR to assess relative steady state mRNA levels for some of the genes of interest identified by SCOTS-array. We chose five genes with high baseline signals and difference in detection signal by SCOTS-array analysis. These included SPA0410 (*eutB*), encoding an ethanolamine ammonia lysase; SPA1451 (*sseE*), encoding a secreted effector protein and located within SPI-2; SPA2748 (*spaP*), encoding a secreted protein located within SPI-1; SPA3315 (*yheL*), encoding a sulfur transfer complex subunit; and SPA3373, a putative cytoplasmic protein. We also assessed SPA3294 transcript levels, encoding ribosomal protein L5, as a representative housekeeping gene. We performed RT-qPCR analyses using blood collected at the initial clinical encounter and immediately preserved in TRIzol. We had sufficient sample to perform RT-qPCR on initial blood samples of SCOTS patients 1 and 3, and performed additional RT-qPCR analysis on two additional patients bacteremic with *S*. Paratyphi A whose blood samples were not analyzed by SCOTS-cDNA hybridization (patients 4 and 5). As shown in figure 3, we found significantly increased expression of all five analyzed candidate genes *in vivo*, but not for housekeeping gene SPA3294.

## Discussion

*S*. Paratyphi A now accounts for a fifth of all cases of enteric fever in many areas of Asia, and it is therefore a significant and emerging global public health concern. *S*. Paratyphi A *in vivo* biology is difficult to study due to a lack of an animal model that fully replicates human infection. In this study, we used an mRNA/cDNA capture and amplification technique, microarray technology, and quantitative PCR to detect *S*. Paratyphi A transcripts in the blood of bacteremic patients in Bangladesh. We identified a subset of *S*. Paratyphi A genes with higher level of transcription *in vivo* compared to *in vitro* samples, suggesting possible *in vivo* induction of these genes, and we confirmed increased *in vivo* expression of a subset of these genes using RT-qPCR. SCOTS and SCOTS/microarray technologies have previously been used to analyze *S*. Typhimurium and *S*. Typhi gene expression using *ex vivo* models [13], [14], [16], [18], but to our knowledge, no group has used this or a similar technology to assess bacterial gene expression directly in the blood of bacteremic patients, and no group has assessed *S*. Paratyphi A gene expression in various conditions.

We could categorize the 868 *S*. Paratyphi A genes expressed in the blood stream of two or more bacteremic patients into a number of groupings that may reflect transcriptional adaptations of *S*. Paratyphi A to the *in vivo* environment. These include adaptations involved in intra-macrophage survival, for example altering energy metabolism, nutrient acquisition, and resisting both oxidative killing and antimicrobial peptides. Forty-three expressed genes were located within known *Salmonella* Pathogenicity Islands including SPI 1-4, 6, 10, 13, and 16 [12], [31]–[34].

SPI-1 encodes a type III secretion system involved in intestinal epithelial cell invasion [38]. Our identification of SPI-1 gene transcripts in the blood stream of infected humans, including genes of the *sip* and *spa* gene clusters, suggests a potential role for these genes in eukaryotic cell invasion outside of the intestinal epithelium. SPI-2 encodes a type III secretion system (TTSS) that is involved in survival of *Salmonella* within macrophages, including creating and maintaining *Salmonella*-containing vacuoles (SCV) [39]. Among the SPI-2 genes detected in our analyses were TTSS-associated and effector genes contained within the *ssa* and *sse* operons. SPI-3 also contains a number of genes involved in pathogenesis. We detected *in vivo* expression of the *mgtBC* operon, encoding a magnesium transporter, within this island, and magnesium availability is a key signal for *Salmonella* in the intracellular environment [40]. Transcription in this operon is regulated by PhoPQ and is required for survival within macrophages and low Mg^2+^ conditions [40]. We also identified the transcript of the SPI-3-associated gene *misL*, an autotransporter possibly involved in cellular adhesion [41]. Within SPI-4, we detected *in vivo* expression of *siiE*, an adhesin that aids in efficient translocation of SPI-1 effectors through involvement in apical membrane ruffling of epithelial cells [42]. SPI-6 appears to be involved in intra-macrophage survival, including encoding proteins involved in metabolic pathways, nutrient acquisition, and utilization of alternate carbon sources within macrophages [43]. Our identification of *ybeJ* involved in carbohydrate transport and metabolism is consistent with this model. SPI-6 also encodes a number of fimbrial proteins involved in adherence and virulence in a number of *Salmonella* animal models [44], and we identified transcripts of *tcfBC* and *safC*, encoding fimbrial proteins, supporting the proposal that these factors may play a role at various stages of *Salmonella* infection. Faucher *et al.* identified SPI-10 in their application of SCOTS to *S*. Typhi within *ex vivo* macrophages [14], and we also detected a number of transcripts of *S*. Paratyphi A genes within SPI-10, many of which are uncharacterized. Interestingly, SPI-10 is present in *S*. Typhi and *S*. Paratyphi A, but absent from most strains of *S*. Typhimurium and all strains of *S*. Paratyphi B and *S*. Paratyphi C examined to date [45]. We also detected transcripts of three genes (*uxaC*, *exuT*, SPA2997) contained within the conserved portion of SPI-13, and *gtrB*, involved in O-antigen glycosylation, and contained within SPI-16 [31].

We detected mRNA/cDNA of a number of genes expressed under the control of major virulence regulatory systems in *Salmonella*, including the PhoP, RpoS, and SlyA systems involved in intra-macrophage survival and virulence [35], [46], [47]. Within the PhoPQ cascade, we detected transcripts of *phoQ* itself; *lpxO*, a dioxgenase involved in lipid synthesis [20]; *mgtBC*; *mgtA*, a possible pseudogene in *S.* Paratyphi A [36]; *virK* encoding the VirK virulence protein [37]; and *bioBF* involved in biotin synthesis [20]. Within the RpoS regulatory cascade, we detected transcripts of *rpoS* itself, *katE* and *xthA* involved in resistance to oxidative stress [48], [49], and *narZYV* involved in nitrate reduction [48], [50]. Of note, mutations in the *narZ* operon are associated with decreased virulence of *S.* Typhimurium in mice [50]. Within the SlyA-regulatory system also involved in intra-macrophage survival of *Salmonella*, we detected expression of *groEL*, a chaperone protein also regulated by PhoP [40], [51].

Many of the genes that we identified as *in vivo* expressed and potentially differentially expressed (compared to *ex vivo* conditions) are associated with adaptation to the likely nutrient-altered environment of the macrophage. For instance, we detected mRNA of 7 of the 17 genes of the *eut* operon involved in ethanolamine utilization, providing alternate sources of carbon and/or nitrogen [52]. We also detected expression of the *pduBGJK* genes involved in alternate carbon source propanediol utilization [43], and genes involved in citrate and tartrate fermentation, including oxaloacetate decarboxylase genes *oadA* and *oadB* [53]. Of note, *oadA* mutants of *Legionella pneumophila* have impaired replication and survival within macrophages [54]. We detected transcripts of *dmsC*, encoding a dimethyl sulfoxide reductase involved in bacterial survival in anaerobic conditions, similar to that probably encountered within *Salmonella*-containing vacuoles, and genes involved in the response to phosphate-limited conditions, including the phosphate transporter genes *ugpAE* and *pstCA-phoU* [55].

*S*. Paratyphi are becoming increasingly resistant to antimicrobial agents, especially in Asia [4], and all *S*. Paratyphi A strains isolated in this study were resistant to nalidixic acid and intermediately susceptible to ciprofloxacin, despite the fact that no antibiotics were administered prior to collection of blood in this study. In our analysis, we detected mRNA/cDNA of several bacterial genes in *in vivo* samples involved in resistance to a number of antimicrobial agents, including nalidixic acid, novobiocin, tetracycline, and norfloxacin [56]. Mechanisms of resistance encoded by detected genes included alteration of target molecules such as the protein encoded by *bacA* that confers resistance to bacitracin [57], and multidrug efflux systems of the resistance-nodulation-division (RND)-type systems (*acrB*, *sapC*, *yegO/mdtC*) and the major facilitator system (*smvA*, *emrA*) [56], [58].

Of the 1798 *in vivo*-expressed genes identified, 910 had statistically significant differences in signal detection when comparing *in vivo* to *in vitro* samples (approximately 50% of detected transcripts; 22.2% of *S.* Paratyphi A ORFs); a figure in concordance with the 36% identified in an *ex vivo* macrophage model of *S*. Typhi by Faucher *et al*. [13]. 194 were concordantly differentially detected in at least two patients, and 41 in all three patients. Many of these 41 genes are involved in energy metabolism, including *eutEJAB*C, *pduG*, and *acpP*; survival in metal ion limiting conditions including the Mn^2+^ transport-associated gene *mntH*; and biosynthesis of essential molecules, including *thiC* involved in thiamine synthesis. Interestingly, we also identified transcripts of *spaP* at higher levels of detection in all *in vivo* versus *in vitro* samples, and subsequently confirmed this increased expression *in vivo* using RT-qPCR. *spaP* is contained within SPI-1 and is thought to be involved in invasion of epithelial cells. Our results suggest a potential role of SpaP in eukaryotic cell invasion beyond the intestinal epithelium.

In conclusion, we have used a capture-amplification and microarray approach to assess gene expression for a human-restricted pathogen, *S*. Paratyphi A, directly in the blood of infected humans. We detected transcripts of many genes contained within known *Salmonella* pathogenicity islands and genes controlled by the PhoPQ, RpoS and SlyA regulons required for intra-macrophage survival. We detected expression of many genes involved in energy metabolism, nutritional acquisition, protein synthesis, fatty acid and phospholipid metabolism, pathogenesis, transport and binding, regulation, SOS responses, and antimicrobial resistance. These functional categories may reflect bacterial modifications required for survival within infected humans. Of note, we also identified expression of a large number of genes with currently unknown function. We further identified a subset of genes whose transcripts had altered detection in *in vivo* versus *in vitro* samples, suggesting potential regulation of these genes within the human host, and we confirmed induction of a subset of these genes *in vivo*. The variability between patients that we observed may relate in part to the low level of bacterial mRNA present, variations introduced by our capture and amplification technology, differences in infecting strains or growth phase *in vivo*, and differences in bacterial location (intra-macrophage versus extracellular). Despite this variability, our results have given insight into bacterial responses in humans infected with *S*. Paratyphi A, and have identified genes for future analysis, including drug target development. These results suggest that similar approaches may be applied to other pathogens in infected humans and animals.

## Supporting Information

Table S1 Sequences of primers used in this study.
(0.04 MB DOC)

Table S2 *S.* Paratyphi A genes whose mRNA/cDNA was detected in the blood of infected humans in this study.
(0.65 MB XLS)
