# Supplementary material for: Analysis of Salmonella enterica Serotype Paratyphi A Gene Expression in the Blood of Bacteremic Patients in Bangladesh
Source: PLoS Negl Trop Dis. 2010 Dec 7;4(12):e908. doi: 10.1371/journal.pntd.0000908 (PMC2998432; doi:10.1371/journal.pntd.0000908)
Supplement: Table S1 — Sequences of primers used in this study. (0.04 MB DOC) [file pntd.0000908.s001.doc]

**Supplemental table 1: Sequences of primers used in this study**

| **SCOTS Primers** |  |  |
| --- | --- | --- |
| **K9RNA** | GAC ACT CTC GAG ACA TCA CCG GTA CCN NNN NNN NN | |
| **F9RNA** | GCC GGA GCT CTG CAG AAT TCN NNN NNN NN | |
| **K9** | GAC ACT CTC GAG ACA TCA CCG G | |
| **F9** | GCC GGA GCT CTG CAG AAT TC | |
| **pRibDNA_PTA construction** | | |
| **16S rDNA**  Upstream  Downstream | ATTCTATCAGAAGCTTATCATGGCTCAGATTGAACG  CATTTATTAGAAGCTTCACCCCAGTCATGAATCACA | |
| **23S rDNA**  Upstream  Downstream | ATTCTTTCAGAAGCTTAGTCAGAGGCGATGAAGGAC  CATTTATTAGAAGCTTAGGTTAAGCCTCACGGTTCA | |
| **qPCR primers** | Upstream | Downstream |
| **SPA0410** | CATACGATCCGTTCCTGGTG | TTAAGGTTCTGGTCGGCATC |
| **SPA1451** | GCGTCGACATCAGGTGTTTA | CCGCGTAAAAGTCCCAGTAA |
| **SPA2748** | CGTCAGGAACCTGTTTCGTT | ATCGCGATAACCATCCAGAC |
| **SPA3294** | TCCTCGTATCCGTGACTTCC | GCCTTCTTCGTCAGATTTCG |
| **SPA3315** | ACCTTACCTCATTGCGCTTC | CCCTGACGACACTGTCTGAA |
| **SPA3373** | CGATACTGCACGGGATTTTT | CGGCGTACTCTGATGTTCAA |
| **16S rRNA** | AGGCCTTCGGGTTGTAAAGT | GACTCAAGCCTGCCAGTTTC |
